# Nomogram-based prediction models for clinical outcomes in pediatric *RUNX1*::*RUNX1T1*-positive acute myeloid leukemia: a retrospective analysis from AML-CAMS serial trials

**DOI:** 10.3389/fonc.2026.1744352

**Published:** 2026-02-13

**Authors:** Xiaoli Chen, Luyang Zhang, Yangyang Zheng, Tianyuan Hu, Meihui Yi, Ye Guo, Xiaojuan Chen, Yumei Chen, Yao Zou, Li Zhang, Wenyu Yang, Yingchi Zhang, Min Ruan, Xiaofan Zhu

**Affiliations:** 1 State Key Laboratory of Experimental Hematology, National Clinical Research Center for Blood Diseases, Haihe Laboratory of Cell Ecosystem, Institute of Hematology & Blood Diseases Hospital, Chinese Academy of Medical Sciences & Peking Union Medical College, Tianjin, China; 2 Tianjin Institutes of Health Science, Tianjin, China; 3 Department of General Surgery, Beijing Hospital, National Center of Gerontology, Institute of Geriatric Medicine, Chinese Academy of Medical Sciences & Peking Union Medical College, Beijing, China

**Keywords:** pediatric acute myeloid leukemia, *RUNX1::RUNX1T1*, measurable residual disease, nomogram, prognosis

## Abstract

**Objective:**

To identify prognostic factors and develop nomograms predicting short-term mortality and relapse in pediatric *RUNX1::RUNX1T1*-positive AML, thereby enabling individualized risk assessment and optimizing clinical management.

**Methods:**

We retrospectively analyzed 136 pediatric patients with *RUNX1::RUNX1T1*-positive AML who achieved morphologic complete remission (CR) after one induction course under AML-CAMS-2009 or AML-CAMS-2016 regimen. Least absolute shrinkage and selection operator (LASSO) and multivariable Cox regression identified independent predictors of 3-year overall survival (OS) and relapse-free survival (RFS). Nomograms were built from these predictors. Model performance was assessed by time-dependent receiver operating characteristic (ROC) curves, calibration plots, decision curve analysis (DCA), and concordance index (C-index), with internal validation performed by bootstrap resampling.

**Results:**

High-Risk measurable residual disease (MRD), treatment regimen, and diagnostic white blood cell (WBC) group (≥20×10⁹/L vs. <20×10⁹/L) independently predicted OS. For RFS, independent predictors were percentage of bone marrow blasts by flow cytometry (BM blasts [FCM]), extramedullary infiltration (EMI), High-Risk MRD, treatment regimen, and WBC group. Nomograms demonstrated strong discrimination and calibration with superior clinical net benefit versus any single predictor. Nomogram-derived scores stratified patients into prognostically distinct subgroups with significant differences in OS and RFS.

**Conclusions:**

This study established internally validated 3-year OS and RFS nomograms for pediatric *RUNX1::RUNX1T1*-positive AML with excellent discrimination and clinical utility. Prospective multicenter validation is warranted to confirm the robustness and facilitate clinical adoption.

## 1 Introduction

Acute myeloid leukemia (AML) is a clonal hematologic disorder characterized by uncontrolled proliferation and arrested differentiation of myeloid precursors, leading to cytopenias and bone marrow failure (1, 2). Although AML predominantly affects older adults, pediatric incidence is relatively low, with an estimated incidence of seven per million children annually (3, 4). The t (8,21)(q22;q22)/*RUNX1::RUNX1T1* fusion—generated by translocation between *RUNX1* (21q22) and *RUNX1T1* (8q22)—is the most frequent cytogenetic abnormality in AML (5), accounting for 10–15% of pediatric cases (6). This subtype is chemo-sensitive with overall favorable outcomes (4, 7); however, only 45-70% of patients remain disease-free long-term and approximately 30% relapse, making relapse the leading cause of treatment failure and death (8, 9).

Early post-remission identification of patients at high risk for relapse is therefore critical for optimizing consolidation and maintenance strategies. Prior studies have evaluated prognostic factors—age, initial WBC count, bone marrow blast burden, and measurable residual disease (MRD) —with inconsistent findings in children (10–13). We previously showed that a reduction in *RUNX1::RUNX1T1* transcript levels of less than three logs after induction therapy, together with MRD ≥0.01% after two consolidations, independently predicted inferior RFS (RFS; HR = 4.230, P = 0.016) and overall survival (OS; HR = 5.128, P = 0.045) (14). However, previous pediatric studies in this subtype have often been limited by relatively small cohort sizes, heterogeneous endpoints and follow-up durations, and variability in MRD methodologies and treatment strategies across institutions and time periods. Consequently, many reports have focused on identifying individual prognostic factors rather than delivering a clinically deployable prediction model. These gaps underscore the rationale for developing the present nomograms to predict outcomes using routinely available clinical variables and standardized response metrics in pediatric *RUNX1::RUNX1T1*-positive AML. Published evidence indicates that patients who achieve an initial complete remission are unlikely to relapse after three years of sustained remission (15). Consistently, among patients who remained in remission for ≥36 months, the cumulative incidence of relapse at 60 months was reported to be 8%, and relative survival after 3 years in remission was comparable to that of a matched general population (16). Given the clinical decision-making relevance and practical actionability of a 3-year endpoint, we selected it as the primary time horizon for risk assessment and developed nomogram-based models integrating routinely available clinical indicators to predict OS and RFS in this population.

## 2 Materials and methods

### 2.1 Study population and treatment regimens

**Figure 1:**
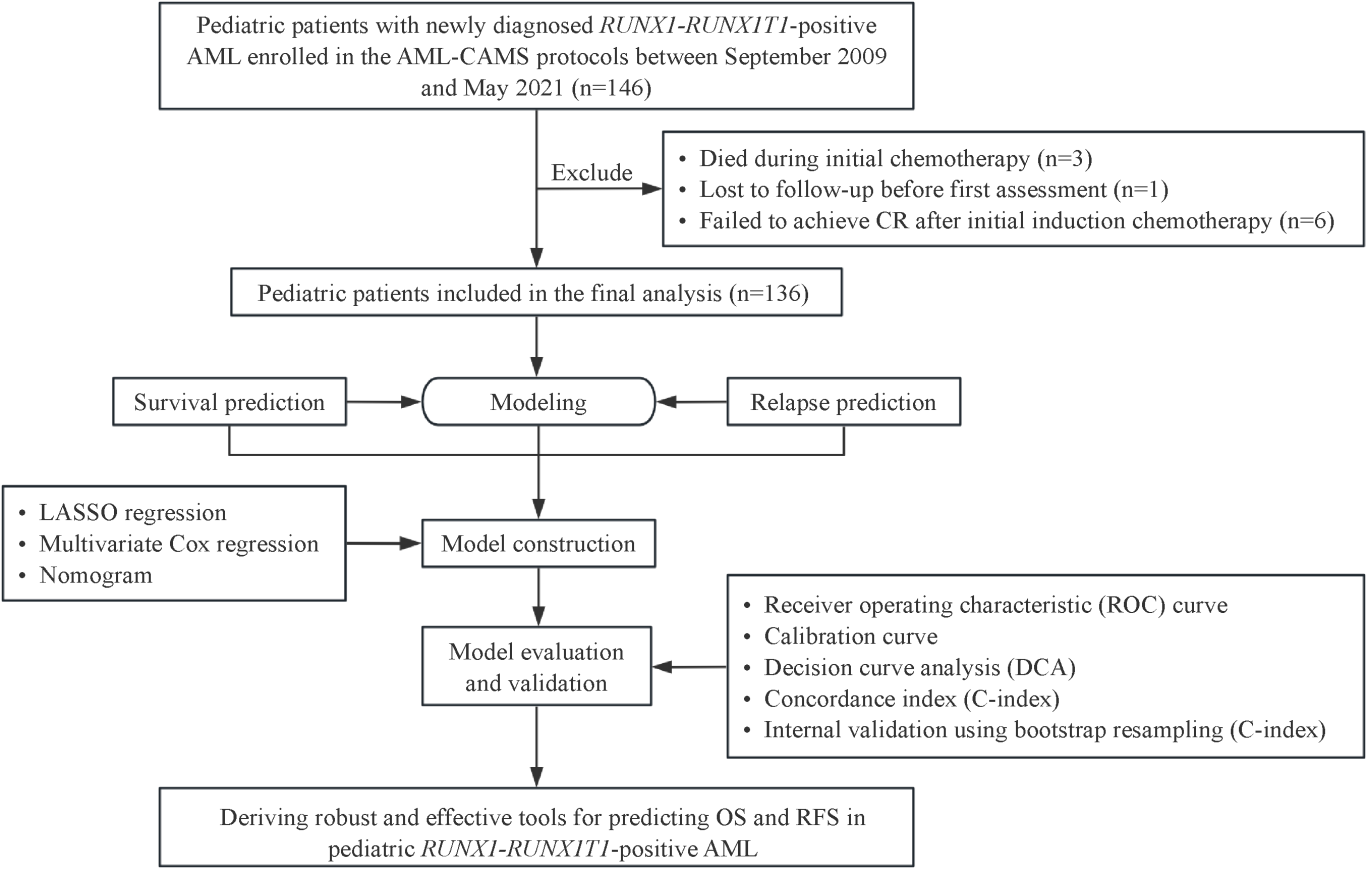
Flowchart illustrating patient screening and the overall study design.

We retrospectively identified consecutive children with *de novo RUNX1::RUNX1T1*-positive AML diagnosed between September 2009 and May 2021 and treated on AML-CAMS serial trials at the Pediatric Blood Disease Center, Blood Diseases Hospital, Chinese Academy of Medical Sciences (CAMS). In total, 146 patients received treatment under AML-CAMS-2009 (NCT03165851) or AML-CAMS-2016 (NCT03173612) protocols. After excluding induction deaths (n = 3), loss to follow-up before the initial assessment (n = 1), and induction refractory disease (n = 6), 136 patients achieving morphological CR after one induction course were included (**Figure 1**).

Both protocols used induction followed by high-dose cytarabine-based consolidation. Regimen assignment was primarily determined by protocol availability during the corresponding trial period based on the calendar time of diagnosis and enrollment, rather than concurrent allocation within the same time period. AML-CAMS-2009 regimen comprised five consolidation cycles; AML-CAMS-2016 used four cycles with reduced-intensity chemotherapy plus dasatinib. Regimen details were reported previously (14, 17). This study was approved by the Institutional Ethics Committee of the Blood Diseases Hospital, CAMS, and performed in compliance with the Declaration of Helsinki. Written informed consent was obtained from guardians. Data were abstracted from existing records and anonymized before analysis.

### 2.2 Clinical variables

Candidate covariates were prespecified *a priori* based on published evidence and biological plausibility in pediatric *RUNX1::RUNX1T1*-positive AML, with an emphasis on clinical relevance and routine availability at diagnosis and early treatment response. Variables were additionally constrained by data completeness and quality in our cohort. We reviewed baseline demographics (age, sex), peripheral blood counts at diagnosis (WBC, hemoglobin, and platelets), presence of extramedullary infiltration (EMI), treatment regimen, and transplantation status. Laboratory and molecular data included *RUNX1::RUNX1T1* transcript quantification by real-time quantitative polymerase chain reaction (RT-qPCR) at diagnosis and subsequent time points, cytogenetics, and *KIT* mutations (14). “High-Risk MRD” was defined as <3-log transcript reduction in *RUNX1::RUNX1T1* after induction and MRD ≥0.01% after two consolidations (14). This combined criterion was prespecified based on our published AML-CAMS MRD dynamics study, in which it was independently associated with inferior RFS and OS (14), and it was also included as a covariate in the prognostic modeling. To more comprehensively capture baseline disease burden and biologically relevant factors potentially associated with clinical outcomes, we additionally incorporated baseline bone marrow (BM) blasts by morphology (BM blasts [Morph]), BM blasts by flow cytometry (BM blasts [FCM]), lactate dehydrogenase (LDH) levels, and RAS-pathway mutations.

Conventional cytogenetic karyotyping and fluorescence *in situ* hybridization (FISH) were routinely performed to screen for and rapidly confirm the t (8,21)(q22;q22) translocation at diagnosis. RT-qPCR was used for baseline quantification of the *RUNX1::RUNX1T1* fusion transcript and for subsequent response assessment and molecular MRD monitoring. A standardized RT-qPCR protocol was implemented. RNA was prepared from BM mononuclear cells, using fresh specimens from AML-CAML-2016 and cryopreserved specimens from AML-CAML-2009, and then reverse-transcribed into cDNA. Quantification of *RUNX1::RUNX1T1* was performed with a TaqMan probe system, with *ABL* serving as the internal reference. Assays were accepted only when *ABL* exceeded 1 × 10^5 copies. The assay sensitivity reached 10 copies per reaction, and transcript levels were presented as the *RUNX1::RUNX1T1*-to-*ABL* copy-number ratio. All molecular analyses were performed in an accredited reference laboratory following standardized protocols. A complex karyotype indicated ≥2 additional clonal chromosomal abnormalities alongside t (8,21). *KIT* and *RAS* pathway mutations were identified by first- or next-generation sequencing in BM.

### 2.3 Follow-up and endpoints

Patients were followed by visits, phone contact, and electronic records. BM aspiration was performed before each chemotherapy course to evaluate treatment response. CR was characterized by <5% blasts in bone marrow smears, no Auer rod, no circulating or extramedullary disease, absolute neutrophil ≥1.0 × 10⁹/L, and platelet count ≥100 × 10⁹/L.

Given the rarity of relapse beyond three years, the observation window was set at three years (15). OS was measured from diagnosis to death due to any cause or last follow-up within this period. RFS was measured from the date of CR to relapse, death, or last follow-up within the same timeframe. Relapse was defined as ≥5% BM blasts (excluding post-chemotherapy regeneration), recurrence of leukemic cells in peripheral blood, or extramedullary infiltration confirmed by morphological or cytogenetic examination.

### 2.4 Statistical analyses

Continuous variables were presented as medians with interquartile ranges (IQRs), and categorical variables were reported as frequencies and proportions. Variables with <10% missingness (excluding outcomes/follow-up) were imputed using the random forest algorithm (mice package, R). Clinical, MICM (morphology, immunophenotyping, cytogenetics, and molecular biology), and treatment variables were analyzed by the least absolute shrinkage and selection operator (LASSO) regression, with optimal penalty parameter (λ) determined by ten-fold cross-validation. Selected variables entered multivariate Cox regression to identify independent prognostic factors, based on which nomograms predicting 1-, 2-, and 3-year OS and RFS were constructed. Model performance was evaluated using receiver operating characteristic (ROC) curves, calibration plots, decision curve analysis (DCA), and concordance index (C-index). Internal validation was performed using 500 bootstrap resamples. Risk scores derived from the nomogram were calculated, and optimal cutoffs identified via X-tile (v3.6.1) to classify patients into high- and low-risk groups. Survival differences were compared using Kaplan-Meier and log-rank tests. In the survival analyses, HSCT was not treated as a censoring event. Patients were followed from diagnosis to the predefined endpoints regardless of HSCT, and relapse or death occurring after HSCT was counted as an event for RFS or OS, respectively. All statistical analyses were conducted using R software (version 4.3.0), and statistical significance was defined as a two-tailed P value below 0.05.

## 3 Results

### 3.1 Cohort characteristics

A total of 136 pediatric patients diagnosed with *RUNX1::RUNX1T1*-positive AML who achieved CR following one course of induction chemotherapy were enrolled in the study. In this cohort, the earliest death occurred 8 months after diagnosis; no induction-related deaths were observed within the first 30 or 60 days after diagnosis. The median diagnostic age was 8 years (IQR, 6–11), and 57.35% were male. During follow-up, 22 patients (16.2%) died and 28 patients (20.6%) relapsed (**Table 1**).

**Table 1:** Comparison of demographic and clinical characteristics according to survival status and relapse status in patients with *RUNX1::RUNX1T1*-positive acute myeloid leukemia (AML).

Characteristics	Total (n = 136)	OS			RFS		
		Alive (*n* = 114)	Dead (n = 22)	P value	Relapse-free (*n* = 108)	Relapsed (n = 28)	P value
Age, y	8.00 (6.00 - 11.00)	8.00 (6.00 - 11.00)	8.00 (6.00 - 10.00)	0.535	8.00 (6.00 - 11.00)	8.50 (6.00 - 10.00)	0.588
Sex				0.111			0.091
Male	78.00 (57.35%)	62.00 (54.39%)	16.00 (72.73%)		58.00 (53.70%)	20.00 (71.43%)	
Female	58.00 (42.65%)	52.00 (45.61%)	6.00 (27.27%)		50.00 (46.30%)	8.00 (28.57%)	
WBC, ×10⁹/L	13.51 (7.43 - 23.11)	11.83 (7.33 - 20.07)	22.31 (14.59 - 32.40)	0.325	12.43 (7.31 - 20.15)	18.14 (9.77 - 29.70)	0.664
WBC group				0.006			0.035
<20×10⁹/L	95.00 (69.85%)	85.00 (74.56%)	10.00 (45.45%)		80.00 (74.07%)	15.00 (53.57%)	
≥20×10⁹/L	41.00 (30.15%)	29.00 (25.44%)	12.00 (54.55%)		28.00 (25.93%)	13.00 (46.43%)	
Hemoglobin, g/L	80.50 (65.00 - 94.00)	81.50 (65.00 - 94.00)	76.50 (60.00 - 94.00)	0.297	80.50 (65.00 - 93.50)	80.00 (60.00 - 94.50)	0.614
Platelet, ×10⁹/L	44.00 (23.50 - 67.00)	45.00 (25.00 - 72.00)	39.00 (19.00 - 65.00)	0.204	44.50 (25.00 - 73.00)	42.50 (20.50 - 60.50)	0.152
LDH, U/L	528.00 (350.75 - 949.50)	506.00 (337.00 - 930.00)	756.50 (411.00 - 1,412.00)	0.187	506.00 (337.00 - 924.50)	756.50 (416.00 - 1,028.00)	0.271
BM blasts (Morph) %	60.00 (47.75 - 75.50)	59.75 (48.50 - 75.00)	71.25 (43.00 - 80.00)	0.814	59.75 (48.75 - 74.00)	64.00 (43.50 - 86.00)	0.630
BM blasts (FCM) %	36.75 (19.85 - 60.15)	34.78 (18.70 - 57.10)	54.40 (20.92 - 68.50)	0.043	34.78 (17.45 - 56.75)	52.77 (20.99 - 67.85)	0.052
EMI				0.615			0.597
No	116.00 (85.29%)	98.00 (85.96%)	18.00 (81.82%)		93.00 (86.11%)	23.00 (82.14%)	
Yes	20.00 (14.71%)	16.00 (14.04%)	4.00 (18.18%)		15.00 (13.89%)	5.00 (17.86%)	
Complex karyotype				0.372			0.301
No	132.00 (97.06%)	110.00 (96.49%)	22.00 (100.00%)		104.00 (96.30%)	28.00 (100.00%)	
Yes	4.00 (2.94%)	4.00 (3.51%)	0.00 (0.00%)		4.00 (3.70%)	0.00 (0.00%)	
X chromosome monosomy				0.597			0.336
No	119.00 (87.50%)	99.00 (86.84%)	20.00 (90.91%)		93.00 (86.11%)	26.00 (92.86%)	
Yes	17.00 (12.50%)	15.00 (13.16%)	2.00 (9.09%)		15.00 (13.89%)	2.00 (7.14%)	
Y chromosome monosomy				0.953			0.379
No	92.00 (67.65%)	77.00 (67.54%)	15.00 (68.18%)		75.00 (69.44%)	17.00 (60.71%)	
Yes	44.00 (32.35%)	37.00 (32.46%)	7.00 (31.82%)		33.00 (30.56%)	11.00 (39.29%)	
9 chromosome monosomy				0.317			0.246
No	131.00 (96.32%)	109.00 (95.61%)	22.00 (100.00%)		103.00 (95.37%)	28.00 (100.00%)	
Yes	5.00 (3.68%)	5.00 (4.39%)	0.00 (0.00%)		5.00 (4.63%)	0.00 (0.00%)	
Any monosomy				0.528			0.940
No	73.00 (53.68%)	60.00 (52.63%)	13.00 (59.09%)		58.00 (53.70%)	15.00 (53.57%)	
Yes	63.00 (46.32%)	54.00 (47.37%)	9.00 (40.91%)		50.00 (46.30%)	13.00 (46.43%)	
*KIT* mutation				0.409			0.359
No	82.00 (60.29%)	67.00 (58.77%)	15.00 (68.18%)		63.00 (58.33%)	19.00 (67.86%)	
Yes	54.00 (39.71%)	47.00 (41.23%)	7.00 (31.82%)		45.00 (41.67%)	9.00 (32.14%)	
*KIT* exon 17 mutation				0.653			0.356
No	87.00 (63.97%)	72.00 (63.16%)	15.00 (68.18%)		67.00 (62.04%)	20.00 (71.43%)	
Yes	49.00 (36.03%)	42.00 (36.84%)	7.00 (31.82%)		41.00 (37.96%)	8.00 (28.57%)	
*KIT* p.D816 mutation				0.228			0.351
No	106.00 (77.94%)	91.00 (79.82%)	15.00 (68.18%)		86.00 (79.63%)	20.00 (71.43%)	
Yes	30.00 (22.06%)	23.00 (20.18%)	7.00 (31.82%)		22.00 (20.37%)	8.00 (28.57%)	
*RAS* mutation				0.923			0.427
No	104.00 (76.47%)	87.00 (76.32%)	17.00 (77.27%)		81.00 (75.00%)	23.00 (82.14%)	
Yes	32.00 (23.53%)	27.00 (23.68%)	5.00 (22.73%)		27.00 (25.00%)	5.00 (17.86%)	
*NRAS* mutation				0.712			0.766
No	109.00 (80.15%)	92.00 (80.70%)	17.00 (77.27%)		86.00 (79.63%)	23.00 (82.14%)	
Yes	27.00 (19.85%)	22.00 (19.30%)	5.00 (22.73%)		22.00 (20.37%)	5.00 (17.86%)	
*KRAS* mutation				0.111			0.065
No	124.00 (91.18%)	102.00 (89.47%)	22.00 (100.00%)		96.00 (88.89%)	28.00 (100.00%)	
Yes	12.00 (8.82%)	12.00 (10.53%)	0.00 (0.00%)		12.00 (11.11%)	0.00 (0.00%)	
HSCT				0.952			0.039
No	118.00 (86.76%)	99.00 (86.84%)	19.00 (86.36%)		97.00 (89.81%)	21.00 (75.00%)	
Yes	18.00 (13.24%)	15.00 (13.16%)	3.00 (13.64%)		11.00 (10.19%)	7.00 (25.00%)	
Treatment regimen				0.008			0.128
AML-CAMS-2009	70.00 (51.47%)	53.00 (46.49%)	17.00 (77.27%)		52.00 (48.15%)	18.00 (64.29%)	
AML-CAMS-2016	66.00 (48.53%)	61.00 (53.51%)	5.00 (22.73%)		56.00 (51.85%)	10.00 (35.71%)	
High-Risk MRD				<0.001			<0.001
No	73.00 (53.68%)	71.00 (62.28%)	2.00 (9.09%)		68.00 (62.96%)	5.00 (17.86%)	
Yes	63.00 (46.32%)	43.00 (37.72%)	20.00 (90.91%)		40.00 (37.04%)	23.00 (82.14%)	

Continuous variables were expressed as medians with interquartile ranges (IQRs), and categorical variables were presented as frequencies and percentages.

Any monosomy was described as the loss of chromosome X, Y, or 9 as detected by conventional cytogenetic analysis. High-Risk MRD was defined as a reduction of the *RUNX1::RUNX1T1* fusion transcript by less than three logs after induction chemotherapy and persistent MRD≥0.01% after two consolidation phases.

OS, overall survival; RFS, relapse-free survival; WBC, white blood cells; LDH, lactate dehydrogenase; BM blasts (Morph) %, percentage of bone marrow blasts by morphological assessment; BM blasts (FCM) %, percentage of bone marrow blasts determined by flow cytometry; EMI, extramedullary infiltration; HSCT, hematopoietic stem cell transplantation; CAMS, Chinese academy of medical sciences; MRD, measurable residual disease monitored by real-time quantitative polymerase chain reaction (RT-qPCR).

At diagnosis, the median WBC count was 13.51×10⁹/L (IQR, 7.43–23.11). Mortality was higher in patients with WBC ≥20×10⁹/L than in those with WBC <20×10⁹/L (54.55% vs 25.44%; P = 0.006). Consistently, a greater proportion of relapsed patients had WBC ≥20×10⁹/L at diagnosis compared with non-relapsed patients (46.43% vs 25.93%; P = 0.035). When stratified by diagnostic WBC, relapse occurred more frequently in the WBC ≥20×10⁹/L group (13/41, 31.71%) than in the WBC <20×10⁹/L group (15/95, 15.79%) (**Supplementary Table 1**). Moreover, among patients who relapsed, post-relapse mortality was higher in the WBC ≥20×10⁹/L group than in the WBC <20×10⁹/L group (**Supplementary Figure 1**). No significant intergroup differences were observed for hemoglobin, platelet count, LDH levels, or BM blasts (Morph). Cytogenetic abnormalities included complex karyotype (2.94%), monosomy X (12.50%), monosomy Y (32.35%), and any monosomy (loss of X, Y, or chromosome 9, 46.32%) without significant differences between survival or relapse groups. *KIT*, *NRAS*, and *KRAS* mutations were detected in 39.71%, 19.85%, and 8.82% of patients, respectively, with no prognostic significance (all P>0.05). Similarly, no marked difference was observed in the frequencies of *KIT* exon 17 or *KIT* p.D816 mutations among groups. Of the entire cohort, 18 patients (13.2%) underwent allogeneic hematopoietic stem cell transplantation (allo-HSCT), with comparable frequencies between survivors and non-survivors (13.16% vs. 13.64%; P>0.05). Additional clinical characteristics of HSCT recipients and HSCT-related details, including indications and timing, are summarized to document real-world HSCT practice in our cohort (**Supplementary Tables 2**, **3**). Treatment regimen and MRD status were key prognostic indicators. The AML-CAMS-2016 regimen was used significantly more often among survivors than non-survivors (53.51% vs. 22.73%; P<0.008), but the difference was not significant between relapse-free and relapsed patients (51.85% vs. 35.71%; P = 0.128). High-Risk MRD was markedly more prevalent in non-survivors vs survivors (90.91% vs. 37.72%; P<0.001) and in relapsed versus relapse-free patients (82.14% vs. 37.04%; P<0.001) (**Table 1**).

### 3.2 Variable selection and nomogram construction

**Figure 2:**
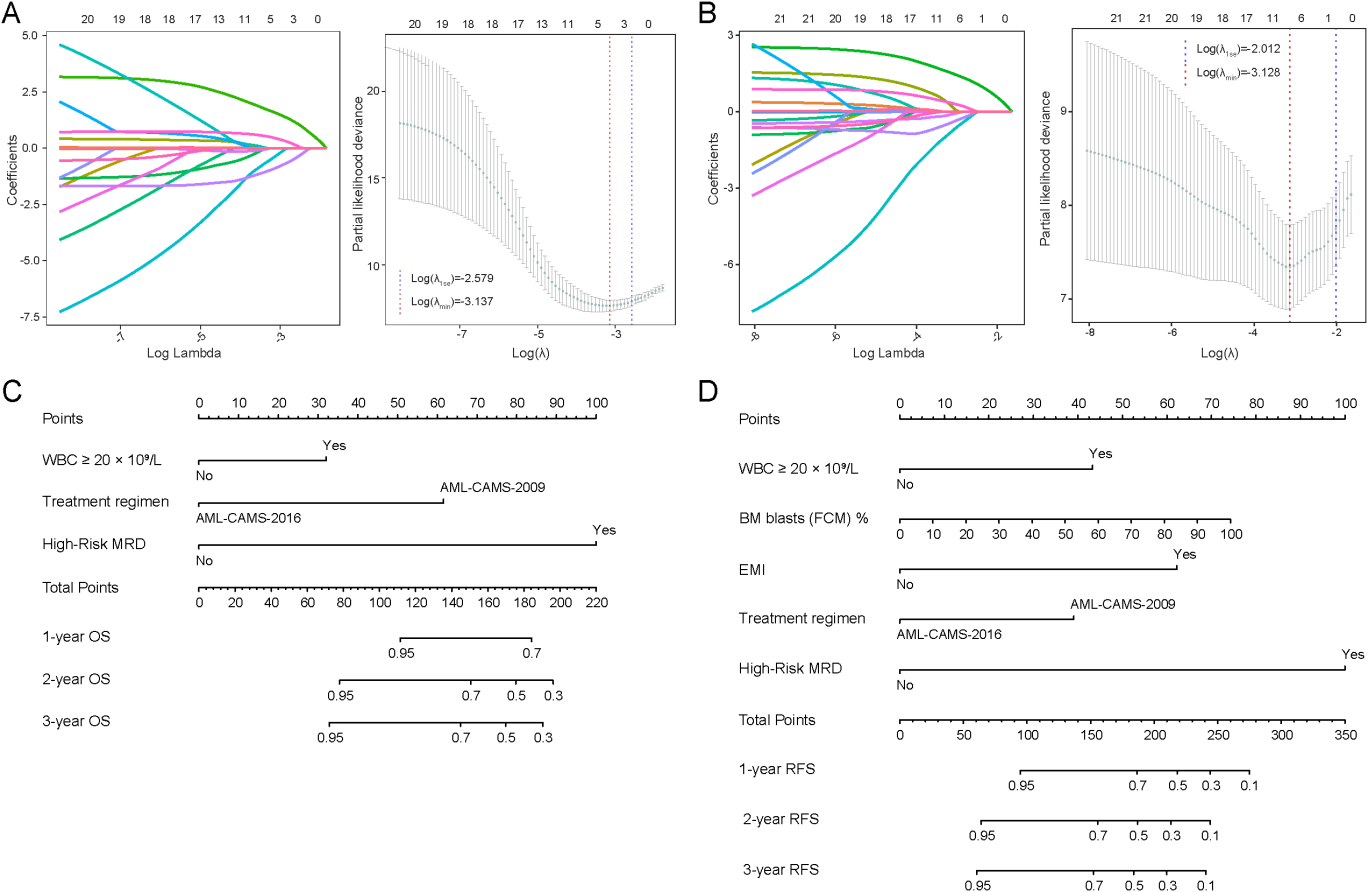
Variable selection using LASSO regression and construction of prognostic nomograms for OS and RFS. LASSO regression coefficient profiles of variables and tuning of the optimal penalty parameter (λ) using ten-fold cross-validation for OS **(A)** and RFS **(B)**.  Nomogram for predicting 1-, 2-, and 3-year OS **(C)** and RFS **(D)** in pediatric patients with *RUNX1-RUNX1T1*-positive-AML. LASSO, least absolute shrinkage and selection operator; λ_1se_, λ under the one-standard-error rule; λmin, λ at minimum cross-validation error.

Based on LASSO regression analysis, key clinical variables associated with OS and RFS in pediatric *RUNX1::RUNX1T1*-positive AML cases were identified. LASSO algorithm with an L1 penalty was employed for variable selection, effectively shrinking the coefficients of non-significant variables to zero, thus eliminating those with minimal contribution to survival prediction. For OS, BM blasts (FCM) %, High-Risk MRD (No/Yes), *KRAS* mutation (No/Yes), treatment regimen (AML-CAMS-2009 vs. AML-CAMS-2016), and WBC group (<20×10⁹/L vs. ≥20×10⁹/L) were selected at λmin (**Figure 2A**). Multivariate Cox analysis confirmed High-Risk MRD, treatment regimen, and WBC group as independent prognostic indicators for OS (**Table 2**), forming the basis of a 1-, 2-, and 3-year OS nomogram (**Figure 2C**). Similarly, the λ min for RFS via LASSO regression selected the following key variables: sex (male vs. female), BM blasts (FCM) %, EMI (No/Yes), High-Risk MRD (No/Yes), *KRAS* mutation (No/Yes), treatment regimen (AML-CAMS-2009 vs. AML-CAMS-2016), and WBC group (<20×10⁹/L vs. ≥20×10⁹/L) (**Figure 2B**). Multivariate Cox regression analysis indicated that BM blasts (FCM), EMI, High-Risk MRD, treatment regimen, and WBC group were independent prognostic factors for RFS in pediatric t (8,21) AML patients (**Table 2**). A nomogram model for 1-year, 2-year, and 3-year RFS was developed and visualized (**Figure 2D**).

**Table 2:** Multivariate Cox proportional hazards regression analysis for overall survival (OS) and relapse-free survival (RFS) in patients with *RUNX1::RUNX1T1*-positive acute myeloid leukemia (AML).

Characteristic	OS		RFS	
	HR (95% CI)	P value	HR (95% CI)	P value
Sex				
Male			Reference	
Female			0.522 (0.215–1.265)	0.150
BM blasts (FCM) %	1.016 (0.996, 1.035)	0.111	1.018 (1.001–1.036)	**0.040**
EMI				
No			Reference	
Yes			5.148 (1.663–15.938)	**0.004**
High-Risk MRD				
No	Reference		Reference	
Yes	13.983 (3.188, 61.341)	**<0.001**	9.633 (3.428–27.073)	**<0.001**
*KRAS* mutation				
No	Reference		Reference	
Yes	0 (0, Inf)	0.998	0 (0, Inf)	0.998
Treatment regimen				
AML-CAMS-2009	Reference		Reference	
AML-CAMS-2016	0.172 (0.061, 0.479)	**0.001**	0.343 (0.144–0.819)	**0.016**
WBC group				
<20×10⁹/L	Reference		Reference	
≥20×10⁹/L	2.928 (1.166, 7.354)	**0.022**	2.553 (1.167–5.586)	**0.019**

High-Risk MRD was defined as a reduction of the *RUNX1::RUNX1T1* fusion transcript by less than three logs after induction chemotherapy and persistent MRD≥0.01% after two consolidation phases.

HR, hazard ratio; CI, confidence interval; BM blasts (FCM) %, percentage of bone marrow blasts determined by flow cytometry; EMI, extramedullary infiltration; MRD, measurable residual disease monitored by real-time quantitative polymerase chain reaction (RT-qPCR); CAMS, Chinese academy of medical sciences; WBC, white blood cells.

Values in bold indicate statistically significant variables (P < 0.05) that were retained and entered into the multivariable model.

In the established nomogram, each category of the prognostic variables was assigned a weighted score proportional to its relative contribution to survival outcomes. The total score, obtained by summing individual points, corresponded to the estimated 1-, 2-, or 3-year OS or RFS probability. A higher total score corresponds to an increased risk and poorer prognosis.

### 3.3 Performance and internal validation

**Figure 3:**
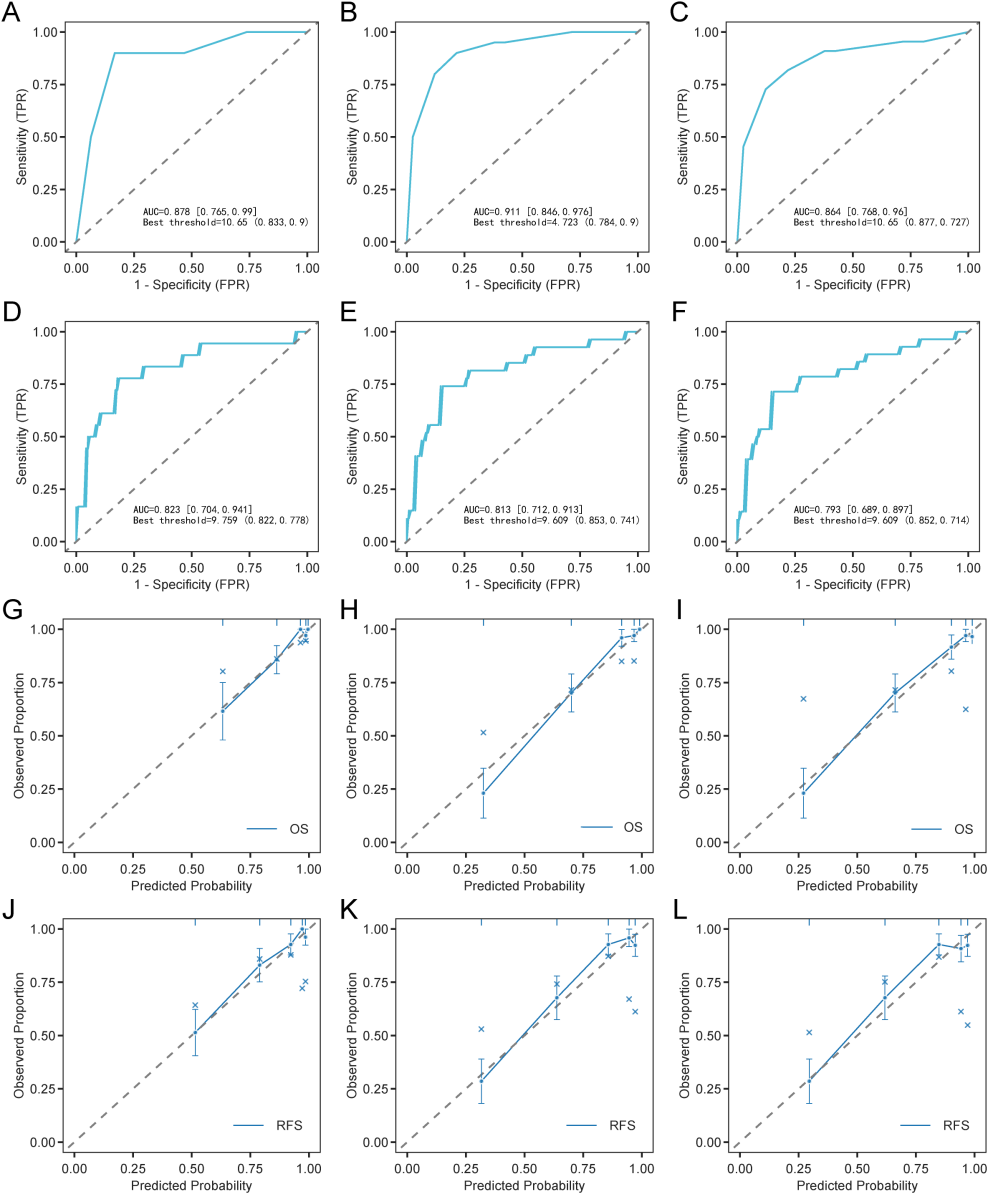
Evaluation of prognostic nomograms for OS and RFS. 1- **(A)**, 2- **(B)**, and 3-year ROC curve **(C)** of nomogram for OS. 1- **(D)**, 2- **(E)**, and 3-year ROC curve **(F)** of nomogram for RFS. 1- **(G)**, 2- **(H)**, and 3-year calibration curve **(I)** of nomogram for OS. 1- **(J)**, 2- **(K)**, and 3-year calibration curve **(L)** of nomogram for RFS.

**Figure 4:**
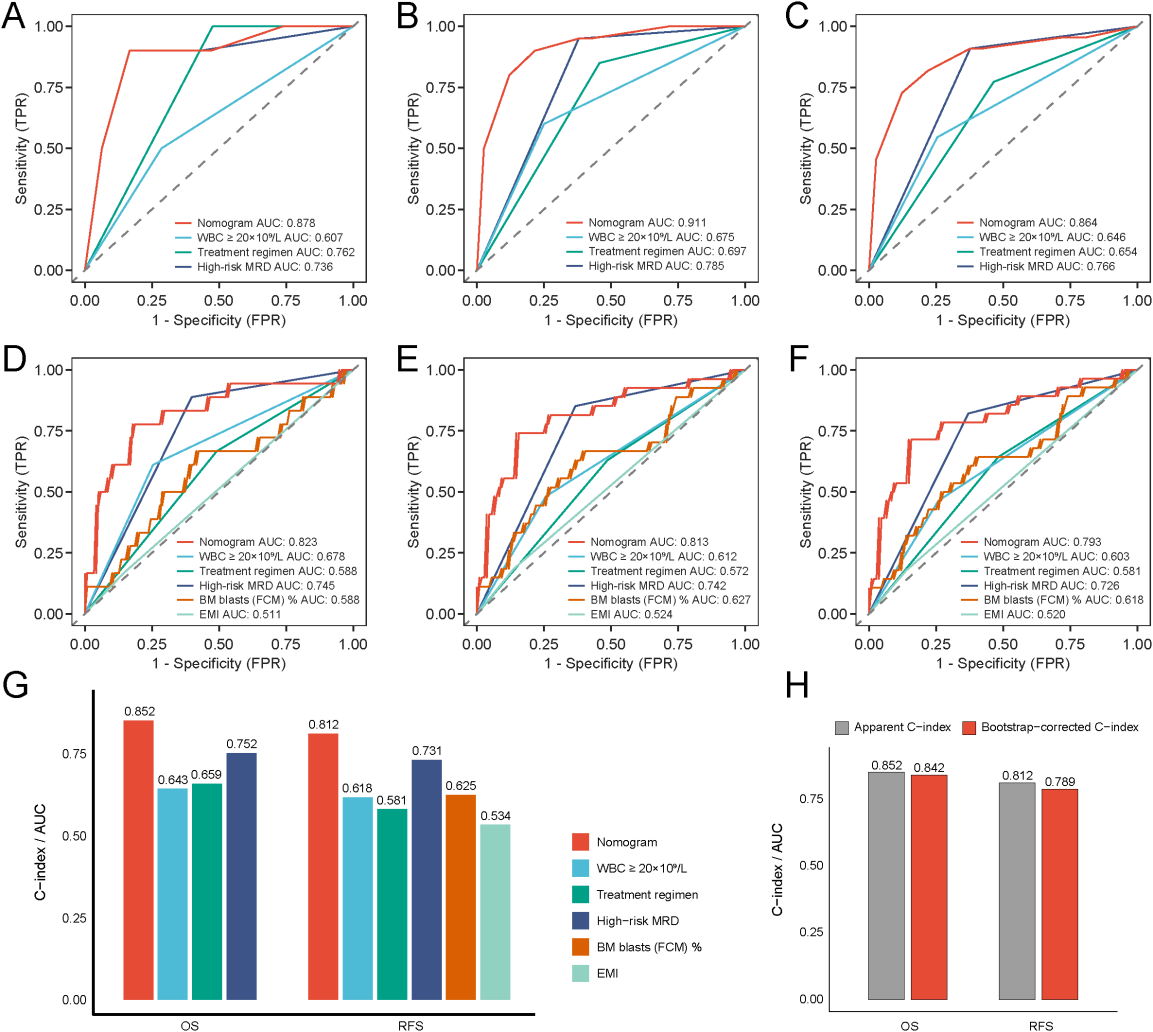
Comparative assessment of the nomogram predictive performance and internal validation of the models. ROC curves of the nomogram and its individual components for predicting 1- **(A)**, 2- **(B)**, and 3-year OS **(C)**. ROC curves of the nomogram and its individual components for predicting 1- **(D)**, 2- **(E)**, and 3-year RFS **(F)**. **(G)** C-index value of the nomogram and its individual components for predicting OS and RFS. **(H)** C-index value using 500 bootstrap resamples of nomogram models for internal validation.

Nomogram performance was systematically evaluated for discrimination, calibration, and clinical applicability. ROC curves showed high area under curve (AUC) values for OS (0.878, 0.911, 0.864 at 1-, 2-, and 3-years) and RFS (0.823, 0.813, 0.793 at 1-, 2-, and 3-years), confirming strong discriminatory ability across different time points (**Figures 3A–F**). Calibration plots showed excellent consistency between predicted and observed outcomes, with calibration curves for 1-, 2-, and 3-year OS and RFS closely aligning with the reference line (**Figures 3G–L**). DCA revealed favorable net clinical benefit across a wide range of threshold probabilities (**Supplementary Figure 1**). Compared with single predictors (OS: High-Risk MRD, treatment regimen, WBC group; RFS: BM blasts (FCM), EMI, High-Risk MRD, treatment regimen, WBC group), the nomograms exhibited consistently higher AUCs, C-index values, and greater clinical net benefit (**Figures 4A–G**; **Supplementary Figure 2**). These findings highlighted the models’ strong predictive performance and practical value for individualized survival estimation in pediatric t (8,21) AML. Bootstrap validation (500 resamples) produced C-indices of 0.842 (OS) and 0.789 (RFS), supporting model robustness (**Figure 4H**).

### 3.4 Nomogram-based risk stratification

**Figure 5:**
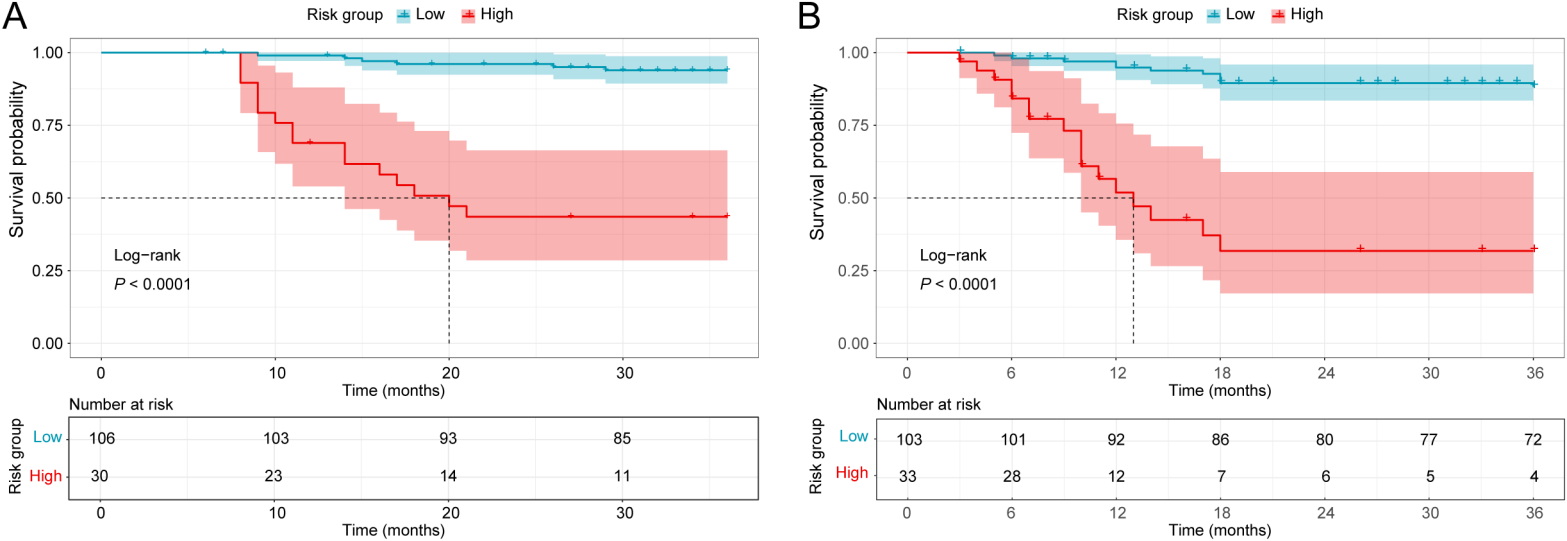
Kaplan-Meier survival curves of different risk groups stratified by nomogram scores. HSCT was not treated as a censoring event; events occurring after HSCT were included in the analysis. **(A)** OS Kaplan-Meier curve. **(B)** RFS Kaplan-Meier curve.

Drawing on nomogram-based scoring results, the optimal cutoff values were determined using X-Tile software to establish a risk stratification system. All participants were stratified into low- and high-risk groups. For OS, scores of 0–160 and 161–194 represented low- and high-risk groups, respectively; for RFS, low-risk group corresponded to scores of 0–240 and high-risk group to scores of 241-350. The Kaplan-Meier survival curves demonstrated that this risk stratification system exhibited excellent discriminatory and stratification abilities (**Figure 5**).

## 4 Discussion

Pediatric *RUNX1::RUNX1T1*-positive AML is considered as a favorable cytogenetic subtype; however, relapse remains the leading cause of treatment failure and death (4, 7–9, 18). Integrating routine clinical data with dynamic treatment responses improves identification of children at high risk of early adverse outcomes. In this study, 136 pediatric patients with *RUNX1::RUNX1T1*-positive AML were retrospectively reviewed and concise prognostic nomograms were developed to predict short-term OS and RFS. LASSO and multivariate Cox regression analyses revealed three independent prognostic factors—High-Risk MRD, treatment regimen, and WBC group—for OS, and five factors—BM blasts (FCM), EMI, High-Risk MRD, treatment regimen, and WBC group—for RFS. The nomograms demonstrated excellent discrimination, calibration, and clinical utility, as confirmed by ROC curves, calibration curves, and DCA analyses. Moreover, the models effectively distinguished patients across high- and low-risk strata, displaying markedly different survival and relapse patterns between groups.

MRD monitoring aims to sensitively detect residual leukemic cells that may lead to relapse and plays a key role in treatment evaluation, relapse prediction, and therapy optimization (19–21). Dynamic changes in molecular MRD levels strongly correlate with prognosis in AML, particularly in cases harboring fusion genes (22–25). Persistent or increasing MRD positivity during or after therapy markedly elevates relapse risk. Previous reports have shown that patients with *RUNX1::RUNX1T1*-positive AML who achieved a ≥2–3 log reduction in transcript levels after induction chemotherapy exhibited a significantly lower relapse risk (26–28). Aligned with previous evidence, our earlier investigation identified that inadequate transcript clearance, defined as a <3-log decline in *RUNX1::RUNX1T1* levels post-induction, was linked to inferior RFS and OS (14). By integrating post-consolidation MRD kinetics, we further delineated a High-Risk MRD pattern, combining suboptimal molecular response (<3-log reduction) with sustained MRD ≥0.01% after two consolidation courses. This MRD profile corresponded to significantly adverse clinical outcomes and an elevated cumulative incidence of relapse (CIR) (14). In this study, High-Risk MRD was incorporated into the prognostic model and further reconfirmed as an independent adverse prognostic factor for 3-year survival and relapse in pediatric t (8,21) AML, exhibiting superior predictive performance compared with other individual variables, except the nomogram model. These results highlight the strong prognostic implication of High-Risk MRD and its potential utility in early risk stratification, personalized therapeutic intervention, and optimizing the timing of HSCT in pediatric AML patients with *RUNX1-RUNX1T1*.

In this study, patients were stratified by WBC counts at diagnosis using a cutoff of 20×10⁹/L. Patients with WBC ≥20×10⁹/L had higher proportions of deaths and relapses, and elevated WBC independently predicted poorer OS and RFS within three years among pediatric t (8,21) AML cases. These findings align with previous studies showing that leukocytosis is generally associated with adverse prognosis, more aggressive disease, and reduced survival (29, 30). For example, one study reported that patients with WBC >10×10⁹/L at induction exhibited significantly worse OS (HR 1.369, 95% CI 1.066–1.759; P = 0.013) and event-free survival (EFS, HR 1.286, 95% CI 1.035–1.599; p = 0.013), as well as increased early death (P<0.001) compared with those without leukocytosis (31). Another study in our center found that WBC ≥50×10⁹/L was linked to higher relapse and early mortality, resulting in markedly poorer outcomes (17). Furthermore, our analyses indicate that the increased mortality observed among patients with diagnostic WBC ≥20×10⁹/L is largely relapse-driven: these patients not only experienced a higher relapse rate but also had poorer survival after relapse. Collectively, these findings underscore the clinical relevance of diagnostic WBC as a readily available marker of relapse susceptibility and post-relapse prognosis in pediatric *RUNX1::RUNX1T1*-positive AML.

Optimization of treatment regimen improved survival and reduced relapse among pediatric t (8,21) AML patients in our study. Compared with AML-CAMS-2009, the AML-CAMS-2016 regimen incorporated the tyrosine kinase inhibitor dasatinib during consolidation with reduced chemotherapy intensity. Given the frequent *KIT* mutations in pediatric *RUNX1-RUNX1T1*-positive AML, dasatinib may improve prognosis via *KIT* pathway inhibition, although prospective clinical studies are warranted to validate its therapeutic effectiveness and safety profile (32, 33). Nevertheless, *KIT* mutation status did not retain independent prognostic significance after multivariable adjustment in our cohort, possibly due to subtype- and VAF-dependent heterogeneity that could not be assessed given the limited sample size and number of events.

Our analysis identified increased BM blasts (FCM) and the occurrence of EMI as independent predictors of three-year relapse in pediatric *RUNX1-RUNX1T1*-positive AML. Within the MICM diagnostic framework, FCM provides quantitative and qualitative assessment of leukemic immunophenotypes. Notably, patients with a higher proportion of BM blasts (FCM) at diagnosis exhibited a significantly increased risk of relapse. This finding supports the concept that the combined interpretation of blast burden and genetic alterations may improve risk stratification and guide more individualized, MRD-adapted management (34, 35). EMI, defined as leukemic blasts infiltration of extramedullary sites such as the liver, spleen, lymph nodes, skin, or central nervous system, occurs more frequently in t (8,21) AML and is associated with inferior outcomes (36–38). Echoing our results, earlier reports confirmed EMI as an independent adverse prognostic factor, with affected patients exhibiting significantly lower three-year RFS compared with those without EMI (68.8 ± 8.8% vs. 88.0 ± 3.4%; P = 0.004) (39). In our cohort, EMI and baseline BM blasts (FCM) were independently associated with RFS but not OS. Clinically, EMI may reflect a more disseminated disease phenotype and potential sanctuary-site involvement, which can facilitate persistence of residual leukemic clones and increase relapse risk (40). Likewise, a higher baseline BM blasts (FCM) likely indicates a higher leukemic burden and may capture biological features link to treatment-tolerant subclones, which may compromise depth of remission and predispose to relapse (41). In contrast, OS represents a more downstream endpoint and is substantially influenced by post-relapse management strategies and interventions (e.g., salvage therapy and HSCT), such that the direct effect of certain baseline risk factors on survival may be attenuated or reshaped by subsequent treatments.

This study has several notable strengths. First, it focuses on the unique subtype of pediatric *RUNX1::RUNX1T1*-positive AML and represents the first study based on a Chinese cohort achieving CR after a single induction course to develop nomograms predicting 3-year mortality and relapse risk. Second, all patients were consecutively enrolled in two national clinical trials (AML-CAMS-2009 and AML-CAMS-2016), ensuring standardized diagnosis, treatment, and follow-up procedures and thereby minimizing study heterogeneity. Third, MRD was dynamically monitored throughout therapy using RT-qPCR, and clinical, MICM, and therapeutic variables were integrated using LASSO and multivariate Cox regression analyses to construct robust prognostic nomograms with strong discriminatory and calibration performance. Finally, internal validation through bootstrap resampling confirmed the models’ stability and clinical applicability, offering a practical tool for individualized prognosis and treatment optimization in pediatric t (8,21) AML.

Notwithstanding its merits, this study is subject to several limitations. First, it was a single-center retrospective study with a modest cohort size, which might introduce selection bias and limit the generalizability of the results. Although strict inclusion and exclusion criteria were applied to minimize bias, the limited number of clinical events (22 deaths and 28 relapses) led to wide confidence intervals for certain variables, such as High-Risk MRD, indicating potential statistical instability. Additionally, as HSCT is a non-randomized therapeutic intervention and was not modeled as a time-dependent exposure, confounding by indication cannot be ruled out. Given that the regimens were implemented in different enrollment periods, temporal changes in supportive care and clinical practice may confound outcome comparisons. Second, the nomograms incorporated only routine clinical data, without including other potential biomarkers. Moreover, genomic profiling was limited largely to *KIT* and *RAS*-pathway alterations, and other mutations and co-mutation patterns were not uniformly available, potentially reducing the detectability of mutation-based prognostic effects. Finally, since all patients were derived from a single Chinese cohort, future studies with larger, multicenter, and ethnically diverse populations are warranted to validate the robustness and broader applicability of the nomograms.

## 5 Conclusion

We developed internally validated nomograms to predict 3-year OS and RFS in children with *RUNX1::RUNX1T1*-positive AML who achieved CR after a single induction cycle. High-Risk MRD, treatment regimen, and WBC group independently predicted OS, while BM blasts (FCM), EMI, high-risk MRD, treatment regimen, and WBC group independently predicted RFS. These clinically parsimonious tools demonstrated strong performance and practical utility for individualized risk assessment and may inform post-remission decision-making. Prospective multicenter validation is warranted.

## Data Availability

The raw data supporting the conclusions of this article will be made available by the authors, without undue reservation.

## References

[1] De Kouchkovsky I Abdul-Hay M . ‘Acute myeloid leukemia: a comprehensive review and 2016 update’. Blood Cancer J. (2016) 6:e441. doi: 10.1038/bcj.2016.50, PMID: 27367478 PMC5030376

[2] Newell LF Cook RJ . Advances in acute myeloid leukemia. Bmj. (2021) 375:n2026. doi: 10.1136/bmj.n2026, PMID: 34615640

[3] Chin PS Bonifer C . Modelling t(8;21) acute myeloid leukaemia - What have we learned? MedComm. (2020) 1:260–9. doi: 10.1002/mco2.30, PMID: 34766123 PMC8491201

[4] Zwaan CM Kolb EA Reinhardt D Abrahamsson J Adachi S Aplenc R . Collaborative efforts driving progress in pediatric acute myeloid leukemia. J Clin Oncol. (2015) 33:2949–62. doi: 10.1200/JCO.2015.62.8289, PMID: 26304895 PMC4567700

[5] Lin S Mulloy JC Goyama S . RUNX1-ETO leukemia. Adv Exp Med Biol. (2017) 962:151–73. doi: 10.1007/978-981-10-3233-2_11, PMID: 28299657

[6] Harrison CJ Hills RK Moorman AV Grimwade DJ Hann I Webb DK . Cytogenetics of childhood acute myeloid leukemia: United Kingdom Medical Research Council Treatment trials AML 10 and 12. J Clin Oncol. (2010) 28:2674–81. doi: 10.1200/JCO.2009.24.8997, PMID: 20439644

[7] Zampini M Tregnago C Bisio V Simula L Borella G Manara E . Epigenetic heterogeneity affects the risk of relapse in children with t(8;21)RUNX1-RUNX1T1-rearranged AML. Leukemia. (2018) 32:1124–34. doi: 10.1038/s41375-017-0003-y, PMID: 29472719

[8] Papaemmanuil E Gerstung M Bullinger L Gaidzik VI Paschka P Roberts ND . Genomic classification and prognosis in acute myeloid leukemia. N Engl J Med. (2016) 374:2209–21. doi: 10.1056/NEJMoa1516192, PMID: 27276561 PMC4979995

[9] Zhou W Li S Wang H Zhou J Li S Chen G . A novel AML1-ETO/FTO positive feedback loop promotes leukemogenesis and Ara-C resistance via stabilizing IGFBP2 in t(8;21) acute myeloid leukemia. Exp Hematol Oncol. (2024) 13:9. doi: 10.1186/s40164-024-00480-z, PMID: 38268050 PMC10807068

[10] Wu J Lu AD Zhang LP Zuo YX Jia YP . Study of clinical outcome and prognosis in pediatric core binding factor-acute myeloid leukemia. Zhonghua Xue Ye Xue Za Zhi. (2019) 40:52–7. doi: 10.3760/cma.j.issn.0253-2727.2019.01.010, PMID: 30704229 PMC7351698

[11] Liu LP Zong SY Zhang AL Ren YY Qi BQ Chang LX . Early detection of molecular residual disease and risk stratification for children with acute myeloid leukemia via circulating tumor DNA. Clin Cancer Res. (2024) 30:1143–51. doi: 10.1158/1078-0432.CCR-23-2589, PMID: 38170574

[12] Sander A Zimmermann M Dworzak M Fleischhack G von Neuhoff C Reinhardt D . Consequent and intensified relapse therapy improved survival in pediatric AML: results of relapse treatment in 379 patients of three consecutive AML-BFM trials. Leukemia. (2010) 24:1422–8. doi: 10.1038/leu.2010.127, PMID: 20535146

[13] Qiu KY Liao XY Li Y Huang K Xu HG Fang JP . Outcome and prognostic factors of CBF pediatric AML patients with t(8;21) differ from patients with inv(16). BMC Cancer. (2023) 23:476. doi: 10.1186/s12885-023-10965-5, PMID: 37231380 PMC10210276

[14] Zhang L Yi M Chen X Guo Y Chen X Chen Y . Measurable residual disease dynamics holding prognostic significance in pediatric patients with RUNX1::RUNX1T1-positive AML: results from AML-CAMS serial trials. Leuk Lymphoma. (2025) 66:1–9. doi: 10.1080/10428194.2025.2548966, PMID: 40828779

[15] Urrutia S Jen WY Sasaki K Wiernik PH Kadia T Daver N . Mortality and relapse dynamics in AML after three years of complete remission. Leuk Lymphoma. (2025) 66:1–6. doi: 10.1080/10428194.2025.2547984, PMID: 40827386

[16] Lim JJ Othus M Shaw CM Russell K Halpern AB Appelbaum JS . Time independent factors that predict relapse in adults with acute myeloid leukemia. Blood Cancer J. (2024) 14:5. doi: 10.1038/s41408-023-00954-z, PMID: 38221523 PMC10788333

[17] Zhang A Liu L Zong S Chang L Chen X Yang W . Pediatric acute myeloid leukemia and hyperleukocytosis with WBC count greater than 50 × 10(9)/L. Int J Hematol. (2023) 118:737–44. doi: 10.1007/s12185-023-03665-0, PMID: 37733171

[18] Klein K Kaspers G Harrison CJ Beverloo HB Reedijk A Bongers M . Clinical Impact of Additional Cytogenetic Aberrations, cKIT and RAS Mutations, and Treatment Elements in Pediatric t(8;21)-AML: Results From an International Retrospective Study by the International Berlin-Frankfurt-Münster Study Group. J Clin Oncol. (2015) 33:4247–58. doi: 10.1200/JCO.2015.61.1947, PMID: 26573082 PMC5321085

[19] Zhu HH Zhang XH Qin YZ Liu DH Jiang H Chen H . MRD-directed risk stratification treatment may improve outcomes of t(8;21) AML in the first complete remission: results from the AML05 multicenter trial. Blood. (2013) 121:4056–62. doi: 10.1182/blood-2012-11-468348, PMID: 23535063

[20] Wei H Liu X Wang Y Lin D Zhou C Liu B . Optimized clinical application of minimal residual disease in acute myeloid leukemia with RUNX1-RUNX1T1. Exp Hematol. (2021) 96:63–72.e3. doi: 10.1016/j.exphem.2021.01.007, PMID: 33524443

[21] Freeman SD Hills RK Virgo P Khan N Couzens S Dillon R . Measurable Residual Disease at Induction Redefines Partial Response in Acute Myeloid Leukemia and Stratifies Outcomes in Patients at Standard Risk Without NPM1 Mutations. J Clin Oncol. (2018) 36:1486–97. doi: 10.1200/JCO.2017.76.3425, PMID: 29601212 PMC5959196

[22] Rashidi A Linden MA DeFor TE Warlick E Bejanyan N Yohe S . History of consolidation is prognostic in acute myeloid leukemia patients undergoing allogeneic hematopoietic cell transplantation in minimal residual disease-negative first complete remission. Am J Hematol. (2017) 92:1032–6. doi: 10.1002/ajh.24834, PMID: 28646534 PMC5734672

[23] Wei AH Iland HJ DiNardo CD Reynolds J . MRD intervention in AML: A new therapeutic horizon. Blood. (2025) 147:13–23. doi: 10.1182/blood.2025029010, PMID: 41105915

[24] Nachmias B Haran A YIsraeli Salman M Stein EM . Measurable residual disease-guided therapy in acute myeloid leukaemia: Practical insights. Br J Haematol. (2025) 207:1192–212. doi: 10.1111/bjh.70038, PMID: 40887043 PMC12512098

[25] Höllein A Jeromin S Meggendorfer M Fasan A Nadarajah N Kern W . Minimal residual disease (MRD) monitoring and mutational landscape in AML with RUNX1-RUNX1T1: a study on 134 patients. Leukemia. (2018) 32:2270–4. doi: 10.1038/s41375-018-0086-0, PMID: 29568097

[26] Boddu P Gurguis C Sanford D Cortes J Akosile M Ravandi F . Response kinetics and factors predicting survival in core-binding factor leukemia. Leukemia. (2018) 32:2698–701. doi: 10.1038/s41375-018-0158-1, PMID: 29884905

[27] Jourdan E Boissel N Chevret S Delabesse E Renneville A Cornillet P . Prospective evaluation of gene mutations and minimal residual disease in patients with core binding factor acute myeloid leukemia. Blood. (2013) 121:2213–23. doi: 10.1182/blood-2012-10-462879, PMID: 23321257

[28] Yang M Wang W Zhang G Qiu S Liu B Mi Y . Clinical characteristics and therapeutic determinants of RUNX1::RUNX1T1 differ from those of CBFB::MYH11 acute myeloid leukemia. Haematologica. (2025) 110:2281–92. doi: 10.3324/haematol.2024.287293, PMID: 40304077 PMC12485314

[29] Djunic I Virijevic M Novkovic A Djurasinovic V Colovic N Vidovic A . Pretreatment risk factors and importance of comorbidity for overall survival, complete remission, and early death in patients with acute myeloid leukemia. Hematology. (2012) 17:53–8. doi: 10.1179/102453312X13221316477651, PMID: 22664041

[30] Greenwood MJ Seftel MD Richardson C Barbaric D Barnett MJ Bruyere H . Leukocyte count as a predictor of death during remission induction in acute myeloid leukemia. Leuk Lymphoma. (2006) 47:1245–52. doi: 10.1080/10428190600572673, PMID: 16923553

[31] Jany J Mikesch JH Wethmar K Baden D Osiaevi I Lenz G . Prognostic impact of WBC kinetics after induction therapy in patients with acute myeloid leukemia (AML). Leuk Lymphoma. (2025) 66:1809–20. doi: 10.1080/10428194.2025.2512029, PMID: 40455055

[32] Al-Harbi S Aljurf M Mohty M Almohareb F Ahmed SOA . An update on the molecular pathogenesis and potential therapeutic targeting of AML with t(8;21)(q22;q22.1);RUNX1-RUNX1T1. Blood Adv. (2020) 4:229–38. doi: 10.1182/bloodadvances.2019000168, PMID: 31935293 PMC6960481

[33] Marcucci G Geyer S Laumann K Zhao W Bucci D Uy GL . Combination of dasatinib with chemotherapy in previously untreated core binding factor acute myeloid leukemia: CALGB 10801. Blood Adv. (2020) 4:696–705. doi: 10.1182/bloodadvances.2019000492, PMID: 32092139 PMC7042984

[34] Ally F Chen X . Acute myeloid leukemia: diagnosis and evaluation by flow cytometry. Cancers (Basel). (2024) 16:3855. doi: 10.3390/cancers16223855, PMID: 39594810 PMC11592599

[35] Schuurhuis GJ Heuser M Freeman S Béné MC Buccisano F Cloos J . Minimal/measurable residual disease in AML: a consensus document from the European LeukemiaNet MRD Working Party. Blood. (2018) 131:1275–91. doi: 10.1182/blood-2017-09-801498, PMID: 29330221 PMC5865231

[36] Byrd JC Weiss RB Arthur DC Lawrence D Baer MR Davey F . Extramedullary leukemia adversely affects hematologic complete remission rate and overall survival in patients with t(8;21)(q22;q22): results from Cancer and Leukemia Group B 8461. J Clin Oncol. (1997) 15:466–75. doi: 10.1200/JCO.1997.15.2.466, PMID: 9053467

[37] Hu GH Lu AD Jia YP Zuo YX Wu J Zhang LP . Prognostic impact of extramedullary infiltration in pediatric low-risk acute myeloid leukemia: A retrospective single-center study over 10 years. Clin Lymphoma Myeloma Leuk. (2020) 20:e813–e20. doi: 10.1016/j.clml.2020.06.009, PMID: 32680776

[38] Gong D Li W Hu LD Shen JL Fang MY Yang QM . Comparison of clinical efficacy of cytarabine with different regimens in postremission consolidation therapy for adult t(8;21) AML patients: A multicenter retrospective study in China. Acta Haematol. (2016) 136:201–9. doi: 10.1159/000448209, PMID: 27640088

[39] Hu G Lu A Wu J Jia Y Zuo Y Ding M . Characteristics and prognosis of pediatric myeloid sarcoma in the cytogenetic context of t(8;21). Pediatr Hematol Oncol. (2021) 38:14–24. doi: 10.1080/08880018.2020.1803462, PMID: 32803999

[40] Bakst RL Tallman MS Douer D Yahalom J . How I treat extramedullary acute myeloid leukemia. Blood. (2011) 118:3785–93. doi: 10.1182/blood-2011-04-347229, PMID: 21795742

[41] François V Alexa SG Jerome T Jean-Emmanuel S Baptiste G Pascale C-L . High levels of CD34+CD38low/–CD123+ blasts are predictive of an adverse outcome in acute myeloid leukemia: a Groupe Ouest-Est des Leucémies Aiguës et Maladies du Sang (GOELAMS) study. Haematologica. (2011) 96:1792–8. doi: 10.3324/haematol.2011.047894, PMID: 21933861 PMC3232261

