# Supplementary material for: Nomogram-based prediction models for clinical outcomes in pediatric RUNX1::RUNX1T1-positive acute myeloid leukemia: a retrospective analysis from AML-CAMS serial trials
Source: Front Oncol. 2026 Feb 13;16:1744352. doi: 10.3389/fonc.2026.1744352 (PMC12945770; doi:10.3389/fonc.2026.1744352)
Supplement: Supplementary file 1 [file DataSheet1.docx]

**Supplementary materials**

**Supplementary Table 1.** Relapse patterns and survival outcomes stratified by diagnostic white blood cell (WBC) group in pediatric *RUNX1::RUNX1T1*-positive AML.

**Supplementary Table 2.** Clinical characteristics according to survival status and relapse status in *RUNX1-RUNX1T1*-positive acute myeloid leukemia (AML) patients undergoing hematopoietic stem cell transplantation (HSCT).

**Supplementary Table 3.** Hematopoietic stem cell transplantation (HSCT)-related clinical details (indications, timing and disease status at HSCT) in pediatric *RUNX1::RUNX1T1*-positive acute myeloid leukemia (AML) patients (n=18).

**Supplementary Figure 1** Relapse patterns and survival distribution stratified by diagnostic WBC group in pediatric *RUNX1::RUNX1T1*-positive AML.

**Supplementary Figure 2** Evaluation of prognostic nomograms for OS and RFS.

**Supplementary Figure 3** Comparative assessment of the nomogram predictive performance.

Supplementary Table 1. Relapse patterns and survival outcomes stratified by diagnostic white blood cell (WBC) group in pediatric *RUNX1::RUNX1T1*-positive AML.

| WBC group | Total  (n = 136) | Relapse  (*n* = 28) | Relapse-free (*n* = 108) | | Relapse (*n* = 28) | |
| --- | --- | --- | --- | --- | --- | --- |
|  |  |  | Alive (*n* = 106) | Dead (*n* = 2) | Alive (*n* = 8) | Dead (*n* = 20) |
| <20×10⁹/L | 95 (69.85%) | 15 (15.79%) | 79 (74.53%) | 1 (50.00%) | 6 (75.00%) | 9 (45.00%) |
| ≥20×10⁹/L | 41 (30.15%) | 13 (31.71%) | 27 (25.47%) | 1 (50.00%) | 2 (25.00%) | 11 (55.00%) |

Supplementary Table 2. Clinical characteristics according to survival status and relapse status in *RUNX1-RUNX1T1*-positive acute myeloid leukemia (AML) patients undergoing hematopoietic stem cell transplantation (HSCT).

| Characteristics | Total  (n = 18) | OS | | RFS | |
| --- | --- | --- | --- | --- | --- |
|  |  | Alive (*n* = 15) | Dead (n = 3) | Relapse-free (*n*  = 11) | Relapsed (n = 7) |
| Age, y | 9.00 (5.00-11.00) | 8.00 (5.00-11.00) | 10.00 (4.00-13.00) | 8.00 (5.00-11.00) | 10.00 (4.00-13.00) |
| Sex |  |  |  |  |  |
| Male | 10 (55.56%) | 9 (60.00%) | 1 (33.33%) | 7 (63.64%) | 3 (42.86%) |
| Female | 8 (44.44%) | 6 (40.00%) | 2 (66.67%) | 4 (36.36%) | 4 (57.14%) |
| WBC, ×10⁹/L | 13.54 (9.61-20.07) | 13.40 (9.61-26.84) | 13.69 (9.16-16.50) | 14.40 (10.30–23.45) | 11.20 (9.77–15.09) |
| WBC group |  |  |  |  |  |
| <20×10⁹/L | 13 (72.22%) | 10 (66.67%) | 3 (100.00%) | 7 (63.64%) | 6 (85.71%) |
| ≥20×10⁹/L | 5 (27.78%) | 5 (33.33%) | 0 (0.00%) | 4 (36.36%) | 1 (14.29%) |
| Hemoglobin, g/L | 83.00 (73.00-97.00) | 83.00 (71.00-100.00) | 83.00 (82.00-93.00) | 95.00 (73.00–103.50) | 82.00 (77.50–83.00) |
| Platelet, ×10⁹/L | 35.00 (21.00-65.00) | 39.00 (22.00-81.00) | 19.00 (18.00-26.00) | 60.00 (29.00–97.50) | 23.00 (20.00–30.00) |
| LDH, U/L | 875.50 (435.00-1430.00) | 832.00 (390.00-1311.00) | 2694.00 (781.00-3926.00) | 919.00 (408.50–1370.50) | 832.00 (613.50–1847.00) |
| BM blasts (Morph) % | 75.50 (58.50-89.00) | 74.00 (57.00-90.00) | 80.00 (58.50-84.00) | 71.50 (54.50–78.75) | 84.00 (70.00–89.50) |
| BM blasts (FCM) % | 58.57 (19.00-67.20) | 57.10 (13.10-63.40) | 68.50 (19.00-75.06) | 34.60 (12.20–58.57) | 67.20 (62.55–70.66) |
| EMI |  |  |  |  |  |
| No | 14 (77.78%) | 11 (73.33%) | 3 (100.00%) | 7 (63.64%) | 7 (100.00%) |
| Yes | 4 (22.22%) | 4 (26.67%) | 0 (0.00%) | 4 (36.36%) | 0 (0.00%) |
| Complex karyotype |  |  |  |  |  |
| No | 18 (100.00%) | 15 (100.00%) | 3 (100.00%) | 11 (100.00%) | 7 (100.00%) |
| Yes | 0 (0.00%) | 0 (0.00%) | 0 (0.00%) | 0 (0.00%) | 0 (0.00%) |
| X chromosome monosomy |  |  |  |  |  |
| No | 18 (100.00%) | 15 (100.00%) | 3 (100.00%) | 11 (100.00%) | 7 (100.00%) |
| Yes | 0 (0.00%) | 0 (0.00%) | 0 (0.00%) | 0 (0.00%) | 0 (0.00%) |
| Y chromosome monosomy |  |  |  |  |  |
| No | 10 (55.56%) | 8 (53.33%) | 2 (66.67%) | 5 (45.45%) | 5 (71.43%) |
| Yes | 8 (44.44%) | 7 (46.67%) | 1 (33.33%) | 6 (54.55%) | 2 (28.57%) |
| 9 chromosome monosomy |  |  |  |  |  |
| No | 18 (100.00%) | 15 (100.00%) | 3 (100.00%) | 11 (100.00%) | 7 (100.00%) |
| Yes | 0 (0.00%) | 0 (0.00%) | 0 (0.00%) | 0 (0.00%) | 0 (0.00%) |
| Any monosomy |  |  |  |  |  |
| No | 10 (55.56%) | 8 (53.33%) | 2 (66.67%) | 5 (45.45%) | 5 (71.43%) |
| Yes | 8 (44.44%) | 7 (46.67%) | 1 (33.33%) | 6 (54.55%) | 2 (28.57%) |
| *KIT* mutation |  |  |  |  |  |
| No | 6 (33.33%) | 5 (33.33%) | 1 (33.33%) | 3 (27.27%) | 3 (42.86%) |
| Yes | 12 (66.67%) | 10 (66.67%) | 2 (66.67%) | 8 (72.73%) | 4 (57.14%) |
| *KIT* exon 17 mutation |  |  |  |  |  |
| No | 7 (38.89%) | 6 (40.00%) | 1 (33.33%) | 3 (27.27%) | 4 (57.14%) |
| Yes | 11 (61.11%) | 9 (60.00%) | 2 (66.67%) | 8 (72.73%) | 3 (42.86%) |
| *KIT* p.D816 mutation |  |  |  |  |  |
| No | 10 (55.56%) | 9 (60.00%) | 1 (33.33%) | 6 (54.55%) | 4 (57.14%) |
| Yes | 8 (44.44%) | 6 (40.00%) | 2 (66.67%) | 5 (45.45%) | 3 (42.86%) |
| *RAS* mutation |  |  |  |  |  |
| No | 13 (72.22%) | 10 (66.67%) | 3 (100.00%) | 6 (54.55%) | 7 (100.00%) |
| Yes | 5 (27.78%) | 5 (33.33%) | 0 (0.00%) | 5 (45.45%) | 0 (0.00%) |
| *NRAS* mutation |  |  |  |  |  |
| No | 13 (72.22%) | 10 (66.67%) | 3 (100.00%) | 6 (54.55%) | 7 (100.00%) |
| Yes | 5 (27.78%) | 5 (33.33%) | 0 (0.00%) | 5 (45.45%) | 0 (0.00%) |
| *KRAS* mutation |  |  |  |  |  |
| No | 18 (100.00%) | 15 (100.00%) | 3 (100.00%) | 11 (100.00%) | 7 (100.00%) |
| Yes | 0 (0.00%) | 0 (0.00%) | 0 (0.00%) | 0 (0.00%) | 0 (0.00%) |
| Treatment regimen |  |  |  |  |  |
| AML-CAMS-2009 | 6 (33.33%) | 4 (26.67%) | 2 (66.67%) | 3 (27.27%) | 3 (42.86%) |
| AML-CAMS-2016 | 12 (66.67%) | 11 (73.33%) | 1 (33.33%) | 8 (72.73%) | 4 (57.14%) |
| High-Risk MRD |  |  |  |  |  |
| No | 5 (27.78%) | 4 (26.67%) | 1 (33.33%) | 3 (27.27%) | 2 (28.57%) |
| Yes | 13 (72.22%) | 11 (73.33%) | 2 (66.67%) | 8 (72.73%) | 5 (71.43%) |

Continuous variables were expressed as medians with interquartile ranges (IQRs), and categorical variables were presented as frequencies and percentages.

Any monosomy was described as the loss of chromosome X, Y, or 9 as detected by conventional cytogenetic analysis. High-Risk MRD was defined as a reduction of the *RUNX1::RUNX1T1* fusion transcript by less than three logs after induction chemotherapy and persistent MRD≥0.01% after two consolidation phases.

Abbreviations: OS, overall survival; RFS, relapse-free survival; WBC, white blood cells; LDH, lactate dehydrogenase; BM blasts (Morph) %, percentage of bone marrow blasts by morphological assessment; BM blasts (FCM) %, percentage of bone marrow blasts determined by flow cytometry; EMI, extramedullary infiltration; HSCT, hematopoietic stem cell transplantation; CAMS, Chinese academy of medical sciences; MRD, measurable residual disease monitored by real-time quantitative polymerase chain reaction (RT-qPCR).

Supplementary Table 3. Hematopoietic stem cell transplantation (HSCT)-related clinical details (indications, timing and disease status at HSCT) in pediatric *RUNX1::RUNX1T1*-positive acute myeloid leukemia (AML) patients (n = 18).

| Patient ID | Treatment regimen | Survival status | Relapse status | Indications for HSCT | Disease status at HSCT | Time from diagnosis to HSCT (months) |
| --- | --- | --- | --- | --- | --- | --- |
| Patient #1 | AML-CAMS-2009 | Dead | Relapse | Relapse | After completion of therapy | 16 |
| Patient #2 | AML-CAMS-2009 | Alive | Relapse | Relapse | After completion of therapy | 12 |
| Patient #3 | AML-CAMS-2009 | Dead | Relapse | Relapse | After completion of therapy | 17 |
| Patient #4 | AML-CAMS-2009 | Alive | Relapse-free | Physician decision | Cons 5 | 8 |
| Patient #5 | AML-CAMS-2009 | Alive | Relapse-free | Matched sibling donor | Cons 2 | 3 |
| Patient #6 | AML-CAMS-2009 | Alive | Relapse-free | High-risk MRD | Cons 2 | 3 |
| Patient #7 | AML-CAMS-2016 | Dead | Relapse | Relapse | Cons 4 | 12 |
| Patient #8 | AML-CAMS-2016 | Alive | Relapse | Relapse | four months after Cons 4, during oral dasatinib therapy | 12 |
| Patient #9 | AML-CAMS-2016 | Alive | Relapse | Relapse | After completion of therapy | 21 |
| Patient #10 | AML-CAMS-2016 | Alive | Relapse-free | High-risk MRD | After completion of therapy | 16 |
| Patient #11 | AML-CAMS-2016 | Alive | Relapse-free | High-risk MRD | two months after Cons 4, during oral dasatinib therapy | 7 |
| Patient #12 | AML-CAMS-2016 | Alive | Relapse | Relapse | After completion of therapy | 18 |
| Patient #13 | AML-CAMS-2016 | Alive | Relapse-free | High-risk MRD | three months after Cons 4, during oral dasatinib therapy | 8 |
| Patient #14 | AML-CAMS-2016 | Alive | Relapse-free | High-risk MRD | three months after Cons 4, during oral dasatinib therapy | 10 |
| Patient #15 | AML-CAMS-2016 | Alive | Relapse-free | High-risk MRD | two months after Cons 4, during oral dasatinib therapy | 6 |
| Patient #16 | AML-CAMS-2016 | Alive | Relapse-free | High-risk MRD | After Cons 2 | 6 |
| Patient #17 | AML-CAMS-2016 | Alive | Relapse-free | High-risk MRD | two months after Cons 4, during oral dasatinib therapy | 8 |
| Patient #18 | AML-CAMS-2016 | Alive | Relapse-free | Physician decision | four months after Cons 4, during oral dasatinib therapy | 10 |

High-Risk MRD was defined as a reduction of the *RUNX1::RUNX1T1* fusion transcript by less than three logs after induction chemotherapy and persistent MRD≥0.01% after two consolidation phases.

Abbreviations: HSCT, hematopoietic stem cell transplantation; CAMS, Chinese academy of medical sciences; MRD, measurable residual disease monitored by real-time quantitative polymerase chain reaction (RT-qPCR). Cons, consolidation chemotherapy.

**
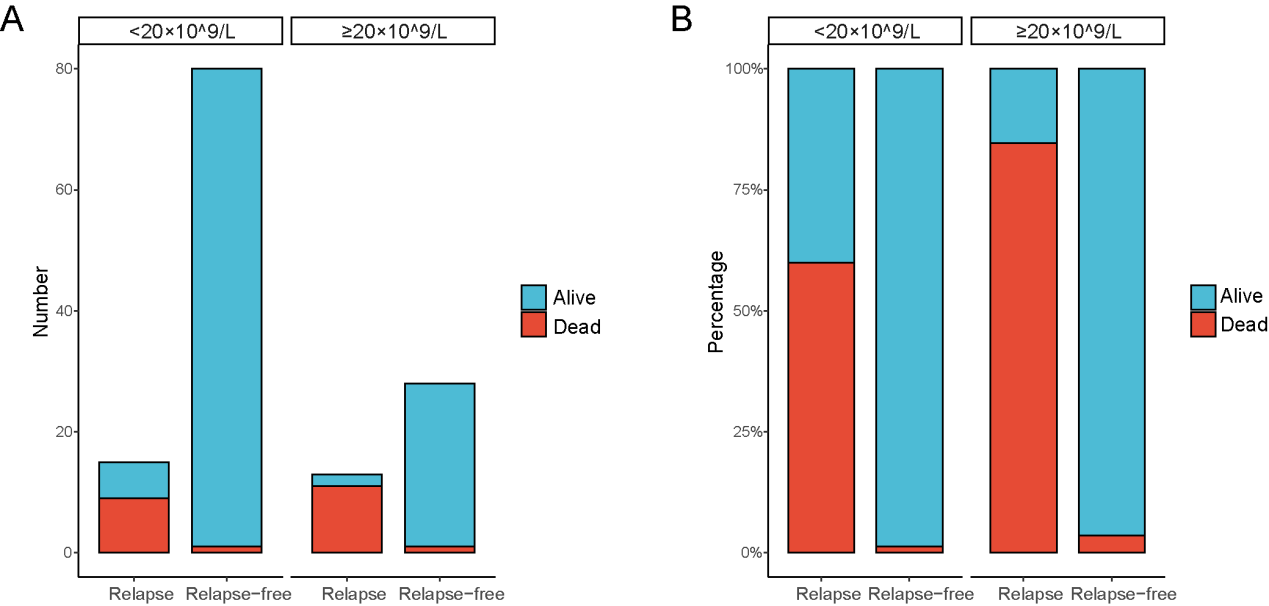
**

**Supplementary Figure 1** Relapse patterns and survival distribution stratified by diagnostic WBC group in pediatric *RUNX1::RUNX1T1*-positive AML. (**A**) The numbers of patients in each category (Alive vs Dead) stratified by diagnostic WBC group and relapse status (Relapse-free vs Relapse). (**B**) The corresponding percentages of patients in each category (Alive vs Dead) stratified by diagnostic WBC group and relapse status.

**
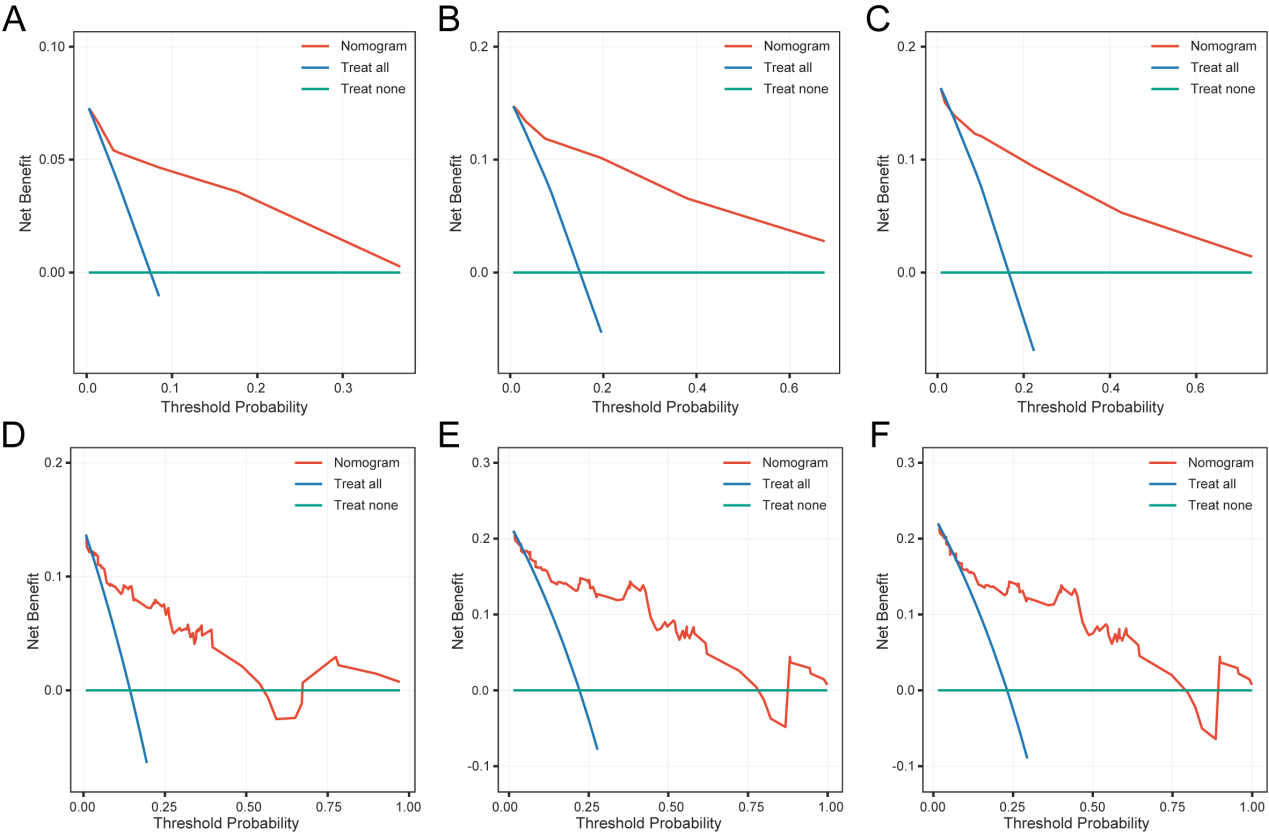
**

**Supplementary Figure 2** Evaluation of prognostic nomograms for OS and RFS. 1- (**A**), 2- (**B**), and 3-year DCA curve (**C**) of nomogram for OS. 1- (**D**), 2- (**E**), and 3-year DCA curve (**F**) of nomogram for RFS. OS, overall survival; RFS, relapse-free survival; DCA, decision curve analysis.

**
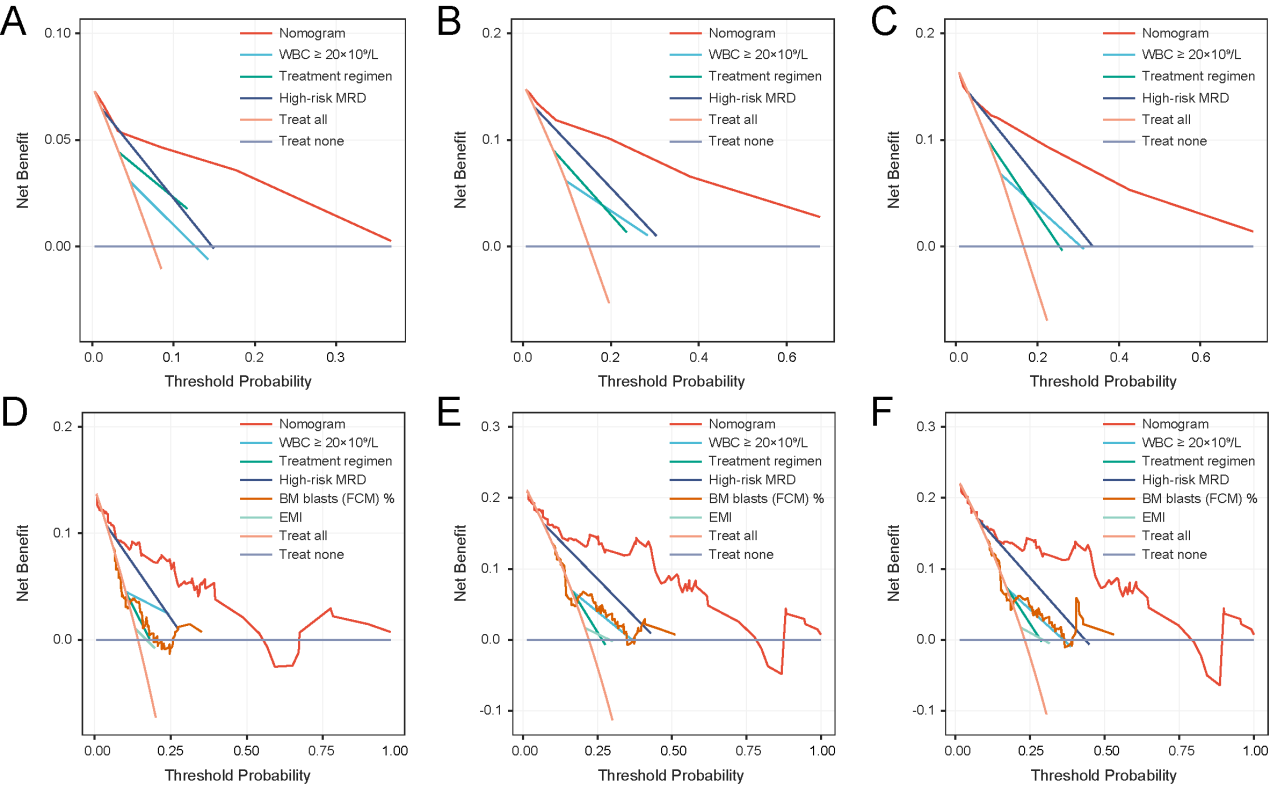
**

**Supplementary Figure 3** Comparative assessment of the nomogram predictive performance. DCA curves of the nomogram and its individual components for predicting 1- (**A**), 2- (**B**), and 3-year OS (**C**). DCA curves of the nomogram and its individual components for predicting 1- (**D**), 2- (**E**), and 3-year RFS (**F**). OS, overall survival; RFS, relapse-free survival; DCA, decision curve analysis; WBC, white blood cells; BM blasts (FCM) %, percentage of bone marrow blasts determined by flow cytometry; EMI, extramedullary infiltration; MRD, measurable residual disease monitored by quantitative real-time polymerase chain reaction (qRT-PCR).
